# Inspection of Hospitals by the Sanitary Commission

**Published:** 1862-12

**Authors:** 


### INSPECTION OF HOSPITALS BY THE SANITARY COMMISSION.

The Sanitary Commission has commenced a special inspection of the General Hospitals of the Army. There are forty-seven in number in the District of Columbia alone, and perhaps as many more in all other parts of the country; containing at this time not less than 50,000 sick and wounded. The Commission propose to keep six Inspectors constantly employed, east and west, and to secure the assistance of the best medical and surgical ability in the country for the work. The Commission express anxiety that this duty be undertaken with the earnest and unselfish purpose of securing for our sick and wounded soldiers thorough and able hospital treatment, by the detection and removal of all defects in administration or professional care susceptible of remedy or improvement. Prof. James P. White and Prof. Thomas F. Rochester of this City have been invited to serve for two terms of a fortnight each, and to designate the time when they can most conveniently render this service.

